# Exosomes: Biomarkers and Therapeutic Targets of Diabetic Vascular Complications

**DOI:** 10.3389/fendo.2021.720466

**Published:** 2021-08-12

**Authors:** Anqi Chen, Hailing Wang, Ying Su, Chunlin Zhang, Yanmei Qiu, Yifan Zhou, Yan Wan, Bo Hu, Yanan Li

**Affiliations:** Department of Neurology, Union Hospital, Tongji Medical College, Huazhong University of Science and Technology, Wuhan, China

**Keywords:** diabetic vascular complications (DVC), exosome, atherosclerosis, diabetic retinopathy (DR), diabetic kidney disease (DKD), stem cells

## Abstract

Diabetic vascular complications (DVC) including macrovascular and microvascular lesions, have a significant impact on public health, and lead to increased patient mortality. Disordered intercellular cascades play a vital role in diabetic systemic vasculopathy. Exosomes participate in the abnormal signal transduction of local vascular cells and mediate the transmission of metabolic disorder signal molecules in distant organs and cells through the blood circulation. They can store different signaling molecules in the membrane structure and release them into the blood, urine, and tears. In recent years, the carrier value and therapeutic effect of exosomes derived from stem cells have garnered attention. Exosomes are not only a promising biomarker but also a potential target and tool for the treatment of DVC. This review explored changes in the production process of exosomes in the diabetic microenvironment and exosomes’ early warning role in DVC from different systems and their pathological processes. On the basis of these findings, we discussed the future direction of exosomes in the treatment of DVC, and the current limitations of exosomes in DVC research.

## Introduction

Diabetes is a chronic disease that threatens public health, and its incidence is increasing yearly. By 2045, the International Diabetes Federation estimates that there will be 700 million diabetic patients worldwide (1). Cardiovascular complications are the leading cause of death among diabetic patients (2). Due to a lack of early, precise diagnostic markers, many patients with diabetes do not receive early diagnosis and treatment of cardiovascular complications (3). At the same time, many symptomatic treatments for vascular complications are expensive and have unstable therapeutic effects (4, 5). Therefore, it is important to find accurate diagnostic markers for DVC and further improve related pathogenesis research.

Even with effective mitigation of existing cardiovascular risk factors, people with type 2 diabetes have approximately twice the risk of developing cardiovascular disease as those without diabetes (6). Studies indicate that more than 50% of diabetic patients die from vascular diseases, such as coronary artery disease, stroke, and peripheral blood vessel disruptions (7). DVC involves macrovascular and microvascular disease (8). Macrovascular disease mainly involves coronary arteries, of which atherosclerosis is the most common (9). Microangiopathy is a vascular complication unique to diabetic patients that mainly affects the vascular changes in the retina and kidney (10). Previously, researchers believed that hemodynamic changes, glucose metabolism disorders, local hypoxia, and vascular dysfunction caused by inflammation were the main causes of the vascular complications of diabetes (11–13). But evidence demonstrates that the connection between systemic cells and organs is also an indispensable part of diabetic vascular damage (14). Exosomes (a type of small membrane vesicles secreted by cells that deliver nucleic acids and proteins) participate in the occurrence of many systemic diseases (15, 16) by regulating inflammation and metabolism (17). They can also mediate the direct communication between vascular endothelial cells and other organs and cells with the help of blood circulation. In addition to the above effects, the latest view is that exosomes are a medium released by stem cells for systemic therapy (18). Exosomes released by various types of stem cells have been found to alleviate the progression of the disease of diabetes (19). Exosomes are considered potential therapeutic targets and valuable biomarkers for the treatment of DVC (20).

This article focuses on the possibility of exosomes as biomarkers in various DVC in human and animal models. We introduce the special pathological changes of exosomes in diabetic microenvironments and their involvement in vascular complications and summarize the value of exosomes in stem cell therapy for DVC.

## Exosome Production Process and Mechanisms

Cell-to-cell and organ-to-organ communication are essential during development, normal physiology, or in pathological conditions. Vesicles secreted by cells under passive or active conditions are imperative to signal transduction pathways. The International Society for Extracellular Vesicles (ISEV) guidelines propose extracellular vesicles (EV) as a general term for particles that are naturally released from cells. These particles are defined by a lipid bilayer and cannot be replicated, that is, they do not contain a functional core. The subtypes of EV include endosome-derived exosomes and plasma membrane-derived ectosomes (microparticles/microvesicles) (21).

Exosome formation begins with the formation of early endosomes when cell membranes recess inward and then continue to mature and form late endosomes. Late endosomes wrap specifically sorted nucleic acids, proteins, and other substances in the cytoplasm to form multiple intraluminal vesicles (ILVS) through inward budding. Multivesicular bodies (MVB) are composed of multiple ILVs in late endocytosis (22–24). MVBs mature process involves a specific sorting machine. Endosomal sorting complex required for transport (ESCRT) is an important driver of MVB membrane formation and rupture (22, 23). The ESCRT system is mainly composed of a family of protein complexes: ESCRT -0, - I, - II, and - III, coupled with the accessory proteins tumor susceptibility gene 101 protein and vacuolar protein sorting-associated protein 4 (25, 26). ESCRT-0 subunits are involved in cargo clustering and the ubiquitination of membrane proteins. ESCRT-I/II/III contribute to plasma membrane budding and exosomes releasing events (27, 28). Cells can also produce ILVs and MVB through lipids, ceramides, or tetrapeptides independent of ESCRT (29–32). The mature MVBs may fuse with lysosomes and degrade due to their ubiquitinated cargo, or they may go toward the plasma membrane and release their contents in the extracellular space with the help of the Rab family and attachment protein receptor (SNARE) complex (33).

**Figure 1:**
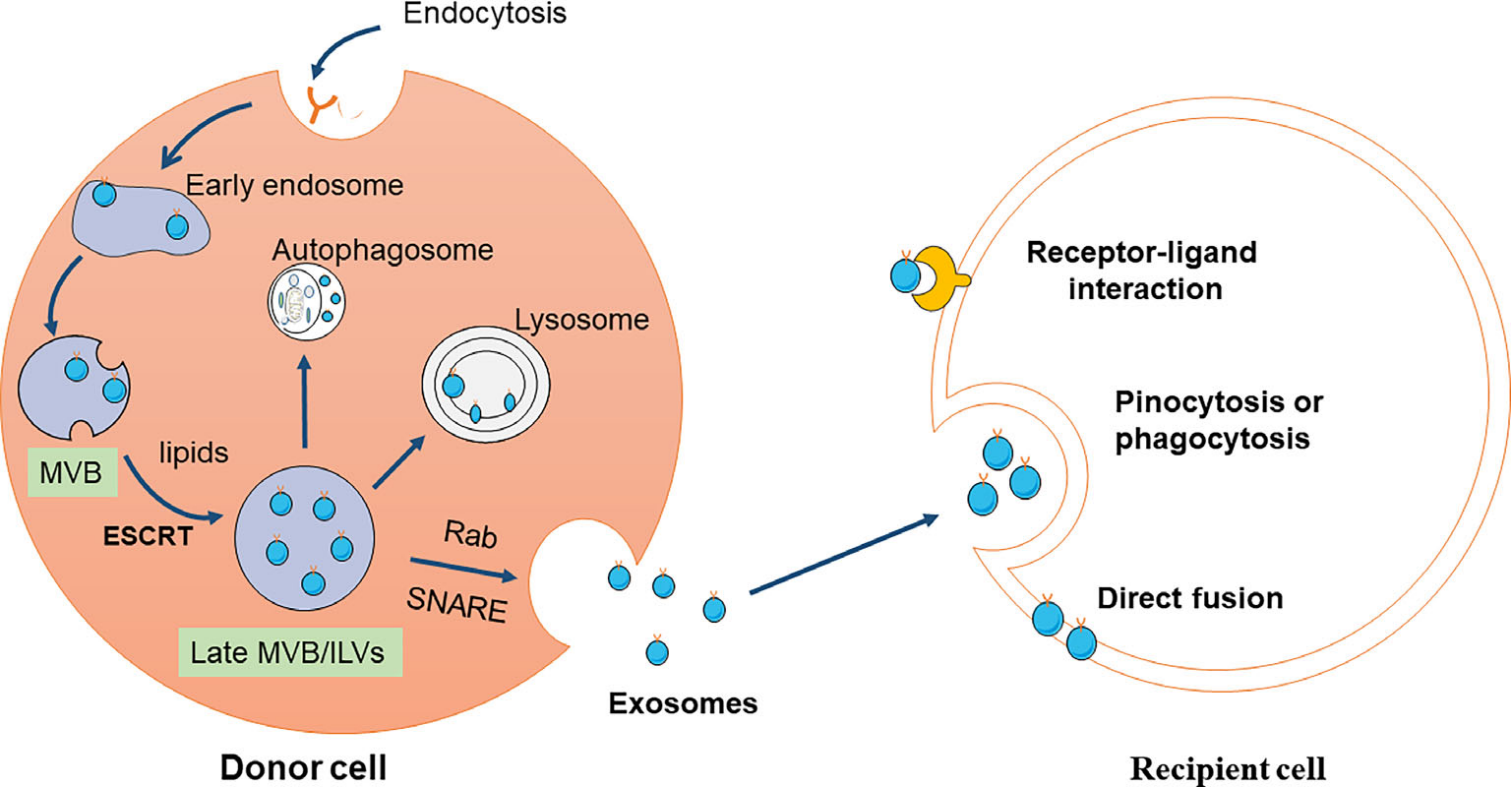
Schematic diagram of exosomes production and reception. Exosomes are originated from the MVBs, referring to double invagination of the plasma membrane. Early sorting endosomes (ESEs) are formed by plasma membrane invagination, which can be fused with the endoplasmic reticulum (ER) and trans-Golgi network (TGN) to produce late sorting endosomes (LSEs). The second invagination in LSE leads to the formation of intraluminal vesicles (ILV). MVBs are formed from LSEs with several ILVs under two mechanisms, ESCRT-dependent and ESCRT-independent pathway. Some MVBs can be degraded by fusion with autophagy or lysosome, while others can be transported to the plasma membrane and released to the extracellular environment. Exosomes are composed of various proteins, lipids, DNA, and RNA. The released exosomes are mainly uptaken by recipient cells through three pathways, including endocytosis, direct fusion, and receptor-ligand interaction.

The exosomes’ primary physiological role is to regulate intercellular communication by transmitting their special cargo, including proteins, lipids, and nucleic acids, in the following three ways. First, exosome membrane proteins can bind to the receptor on the target cell membrane to activate the signal pathways in the target cell. Exosome membrane proteins can be cleaved by some proteases, and the cleaved fragments can bind to cell membrane receptors, thus activating intracellular signaling pathways (34). Third, exosomes directly fuse with the target cell membrane and nonselectively release their contents to regulate target cell signaling (32, 35) (**Figure 1**).

## Effects of Exosomes in Diabetes Mellitus

There is accumulating evidence that exosome levels are elevated in the blood of diabetic individuals and are involved in diabetes-related pathophysiology, including vascular complications, inflammation, and coagulation changes (36–38). In a cross-sectional study of normoglycemic participants and patients with prediabetes or diabetes mellitus, plasma extracellular vesicles in diabetic patients were higher than those in euglycemic control subjects. Exosome concentration was positively correlated with insulin resistance index (HOMA-IR). In vitro, insulin signaling was reduced, and EVs’ secretion increased after the primary neurons long-term exposure to insulin (36). New research has found that autophagy has the same molecular mechanism as exosomes (39, 40). It has been reported that ATG5-ATG12 and ATG16L1 complexes and LC3 have been detected in endosomes and phagosomes (41–43). The function of these autophagy-related proteins is to ensure that the vesicles are acidified and degraded in the lysosome. Using bafilomycin A1 to inhibit the autophagy pathway can prevent the fusion of endosomes and lysosomes, thereby facilitating the release of exosomes (44). Autophagy pathway of multiple cells is inhibited in the diabetic microenvironment, which may increase exosomes in diabetic patients (45–47).

The abnormal exosomes of stem cells are also an essential factor in DVC. Mesenchymal stem cells (MSCs) can be recruited around blood vessels under normal and pathological conditions. They secrete many factors through the exosome pathway to promote the formation of new blood vessels. Therefore, they are considered an essential part of regulating vascular regeneration (48, 49). In aging or diabetic conditions, the production process of MSC exosomes presents significant changes (50). The Jafar Rezaie team exposed human mesenchymal stem cells to diabetic serum for 7 days. It was observed that the levels of exosomal-related genes CD63, Alix, Rab27a, Rab27b and Rab8b increased significantly. In diabetic patients, the activity of acetylcholinesterase is enhanced, the exocrine volume increases and the zeta potential decreases. Ultrastructural examination revealed that more cytosolic lipid vacuoles accumulated in diabetic cells. The reduction in the zeta potential of exosomes is mainly related to the glycosylation of exosomes, such as lactosamine, mannose and N-linked glycans. It indicates that the abnormal glucose and lipid metabolism of stem cells in the diabetic microenvironment may mediate the modification and content alteration of exosomes (51). More importantly, Jafar Rezaie found that the angiogenesis and recovery ability of human bone marrow mesenchymal stem cells decreased after treated with serum from patients with type 2 diabetes. The survival rate of human bone marrow mesenchymal stem cells and their chemotaxis to vascular endothelial growth factor decreased after 7 days of serum culture in patients with type 2 diabetes. After diabetic serum treatment, the expression of cadherin and NG2 in vascular endothelial cells and the uptake capacity of low-density lipoprotein decreased. The cell migration rate and the activity of matrix metalloproteinase-2 and -9 decreased (52). Another interesting study found that metformin, a common drug used to treat diabetes, increased the production of human glioblastoma cell (U87 MG) exosomes. Metformin reduces the activity of U87 MG human glioblastoma cells and inhibits the expression of cancer-promoting genes related to angiogenesis, carcinogenesis and chemoresistance molecules (53). However, previous reports found that the expression of CD63, Alix, and Rab27A decreased in human endothelial progenitor cells (EPCs) cultured in diabetic serum (54). This suggests that the effect of the diabetic microenvironment on exosome production of different types of stem cells may differ.

Evidence shows that inflammatory system disorders in diabetes induce and accelerate various vascular diseases (55–57). The researchers observed that EVs from diabetic patients were preferentially internalized by circulating monocytes. Further studies have shown that co-incubation of diabetic EVs with monocytes could reduce the expression of genes related to apoptosis and oxidative stress in monocytes (36, 58). It also increased the expression of many proinflammatory cytokines (58–60). These findings suggest that EVs in diabetic patients promote monocyte survival and promote monocyte inflammatory activation. Therefore, an abnormal exosome internalization and activation mechanism may be a significant target in the study of diabetic inflammation activation.

## Exosomes Are Involved in Different Vascular Complications

### Atherosclerosis

Atherosclerosis is a disease in which abnormal metabolism or blood coagulation causes thickening and hardening of the arterial walls, resulting in narrowing and obstruction of the vascular lumen (61, 62). Diabetes plays a crucial role in accelerating atherosclerosis, with an augmented inflammatory state, more diffuse atherosclerosis, and larger necrotic core areas in the lesion (63). Endothelial and smooth muscle cell dysfunction, attributing to hyperglycemia and insulin resistance, are the major characteristics of diabetic vasculopathy, favoring a pro-inflammatory/thrombotic state that results in atherothrombosis. In the early stage, lipids or glycoproteins are deposited on the arterial intima, and the damaged endothelium recruits blood-circulating mononuclear cells. These differentiate into macrophages that engulf lipids and transform into foam cells. Subsequently, activated endothelium and macrophages release several chemokines and growth factors, leading to smooth muscle proliferation and extracellular matrix protein synthesis. With the accumulation and denaturation of lipids, fibrous cap tissue necrosis, progressive structural remodeling, and inflammatory cell infiltration, the atheromatous plaques begin to form (62, 64). In recent years, numerous investigations suggest that the exosomes in the diabetic microenvironment promote the progression of atherosclerosis *via* endothelial function, inflammatory pathways, and lipid metabolism (65–67).

Endothelial dysfunction is the basic mechanism of early atherosclerosis in diabetes mellitus. An analysis of patients with atherosclerosis showed that cargo proteins levels in plasma endothelial cell-derived exosomes (EDEs), such as VCAM-1, vWF, PDGF-BB, angiopoietin-1, were significantly higher relative to those of matched control subjects. This is related to the different functions of endothelial cells, such as adhesiveness, antithrombosis, survival and proliferation, transport, metabolism, and the vascular collagen structure (68). Moreover, reduced nitric oxide (NO) bioavailability enables hyperglycemia to impair vascular endothelial function (69). Furchgott and Lgnarro proposed that NO can be used as an endothelial vasodilator to reflect the vascular endothelium function (70, 71). In the early stage of atherosclerosis, NO secretion decreases after vascular endothelial injury, which leads to the increased expression of adhesion molecules, the adhesion of macrophages, LDL oxidation, and smooth muscle proliferation to accelerate atherosclerosis progression (72, 73). Huina Zhang et al. transferred exosomes from the blood of *db/db* diabetic mice into *db/m^+^* non-diabetic mice and found that exosomes can be delivered to the aortic endothelial cells of non-diabetic *db/m+* mice and damage endothelial cell function. Further analysis on exosomal proteins in the endothelial cells of diabetic mouse aortas found that exosome protein signals played a major role in this process. Proteomics analysis showed that arginase 1 in the exosomes of the db/db group was significantly increased (74). The main substrates for NO synthesis are L-arginine and hydroxyl L-arginine. Arginase 1 can specifically degrade NO substrates and reduce NO production in endothelial cells (75). This study demonstrated that lesions in the aortic endothelium could affect other normal cell parts *via* certain regulatory proteins carried by exosomes.

A disordered local microenvironment is an important condition for plaque formation. Exosomes from mature dendritic cells (DCs) are involved in increasing endothelial inflammation and atherosclerosis *via* the NF-κB pathway mediated by tumor necrosis factor-α (TNF-α) located in the exosome membrane (76). Naive and M2-polarized macrophage-derived exosomes, which carry and transfer miR-146b-5p, miR-378-3p, and miR-99a-5p, have a protective role in the suppression of the inflammatory response *via* NF-κB and TNF-α signaling. They also reduce necrotic lesion areas in the atheroma (77).

Additionally, recent studies have revealed that inflammasome activation is one of the pathologic mechanisms of diabetic vascular endothelial dysfunction (78–80). NLRP3 inflammasome is activated in the coronary endothelial cells of early diabetic mice (81). The NLRP3 inflammasome is a multimeric protein complex that mediates caspase-1 activation and the secretion of pro-inflammatory cytokines IL-1β/IL-18 in response to a stimulation (82). Inflammatory body products are not secreted through specific Golgi-mediated direction and transportation, but they are segregated by the membrane and secreted in extracellular vesicles. Exosomes mediate the release of inflammatory body products into the extracellular space (82, 83). In a diabetic mouse model induced by streptozotocin (STZ), endothelial-specific lysosomal acid ceramidase (AC) knockout mice (*Asah1^fl/fl^/EC^cre^*) significantly elevated the formation of NLRP3 inflammasomes and activation in coronary artery ECs (CECs). A metabolic enzyme of ceramide, AC is involved in the transportation of MVBs to lysosomes, thereby regulating the fate of MVBs. In *in vitro* experiments, AC knockout CECS can reduce the interaction between MVB and lysosomes, promote exosome production, increase the release of IL-1β exosomes under high glucose stimulation, and promote an inflammatory reaction, which leads to vascular inflammation and atherosclerosis (84). Therefore, regulating inflammatory responses mediated by exosomes may be an effective way to treat diabetic atherosclerosis.

Improving the plaque stability of advanced plaque and reducing the core of necrosis are the main goals of the current treatment of atherosclerosis. Studies have shown that atherosclerotic plaques in subjects with type 2 diabetes are more likely to rupture. As a hedgehog (hh) signaling molecule, sonic hedgehog (shh) plays a critical role in normal embryonic development and the maintenance of adult vasculature and neovascularization (85). Hh signaling contributes to the progression of atherosclerosis, and its inhibition resulted in a reduction in the plasma cholesterol content. These effects were related to a reduced uptake of modified lipoproteins mediated by scavenger receptors on plaque macrophages (86). Adipocytes are an important endocrine cell involved in controlling appetite and satiety, regulating energy metabolism, and effecting insulin sensitivity (87). Adipocyte dysfunction regulates multiple diabetic complications *via* insulin resistance and tissue inflammation (88). Insulin resistance adipocyte-derived exosomes contribute to vasa vasorum angiogenesis *via* the shh signaling pathway in endothelial cells ex vivo, and further promote plaque burden and plaque vulnerability in diabetic *ApoE-/-* mice (66). Generally, exosomes play an important role in regulating endothelial injury, inflammatory activation, and plaque instability during atherosclerosis development.

## Microvessels

### Diabetic Retinopathy (DR)

DR is a common complication of diabetes. Pathological angiogenesis and leakage of the blood-retinal barrier are characteristic changes in diabetic patients (89). Diabetic retinopathy is usually asymptomatic in its early stages. As the disease progresses, patients experience decreased vision due to macular edema, vitreous hemorrhage, and pathological neovascularization. In severe patients, fibrous tissue proliferation in the vascular scar can cause detachment by pulling the retina (90). Current studies on the molecular mechanisms of DR have revealed the critical role of neurovascular crosstalk disorders in retinal injury (91, 92). As an important carrier of cell crosstalk, the role of exosomes in DR is gradually being recognized.

Müller cells are the most abundant glial cells in the retina, and they play an irreplaceable role in maintaining the blood-retinal barrier and the function of neurons (93). Müller cells have a dual role in DR. On the one hand, high glucose can damage the normal transport function of Müller cells and affect retinal homeostasis (94). On the other hand, the angiogenic factors released by Müller cells and the excessive proliferation of Müller cells are considered to be the central effector cells of the proliferative diabetic retina (95). It is well known that the pathogenesis and progression of diabetes are related to the dysfunction of pancreatic beta cells (96). Tengku Ain Kamalden et al. found that the level of miR-15a in the plasma of diabetic patients is positively correlated with the severity of retinopathy. miR-15a is a crucial molecule that regulates insulin secretion by pancreatic β cells. The exosomes released by pancreatic β cells carry miR-15a into the retina and activate Müller cell apoptosis through the Akt3 pathway (97). In contrast, Zhang Wei’s team found that exosomes isolated from the plasma of diabetic rats can promote the migration and proliferation of Müller cells. In further mechanism studies, it was confirmed that diabetic plasma exosomes enhanced fibronectin and connective tissue growth factor (CTGF) expression in Müller cells, activated Yes-related proteins and jointly promoted the proliferation and fibrosis of Müller cells. These results suggest that exosomes regulate the functions of Müller cells through very complex and delicate pathways. To clarify the specific pathway requires more careful sorting and identification of exosomes.

The retinal pigment epithelium (RPE) is located between the choroid and the retina, forming an external blood-retinal barrier. RPE regulates the translation of epithelial cells and the stability of tight junctions. The regulation of retinal pigment epithelium in diabetic retinopathy is a direction that cannot be ignored (98). Researchers exposed retinal pigment epithelial cell line-19 (ARPE-19) to a high-glucose (HG) medium and detected many exosomes carrying VEGF and ROS. Melanin receptor 5 (MCR5) agonist treatment can significantly reduce the production of ROS and VEGF-carrying exosomes in the retinal pigment epithelium and reduce the angiogenesis caused by ARPE19 induced by high glucose (99). Nevertheless, in another study, Shun Gu et al. found that the release of miR-202-5p-containing exosomes from ARPE19 cells treated with HG can effectively prevent proliferative diabetic retinopathy. The ingestion of these exosomes inhibited the growth, migration and tube formation of human umbilical vein endothelial cells. The miR-202-5p in exosomes can also inhibit endothelial-mesenchymal transition through the TGF/Smad pathway (100).

Vascular inflammation and activation of the complement system play an important role in vascular barrier leakage (101, 102). Complement can be activated by a classical pathway, selective pathway, and lectin pathway. All complement pathways can activate the production of C3/C5 invertase and form a membrane attack complex (MAC) (103). Previous studies in DR patients and diabetic rat models have found that MAC is extensively deposited in retinal endothelial cells, leading to retinal endothelial cell death and increased retinal vessel leakage (104, 105). Huang C et al. found that the content of exosomes in the plasma of diabetic patients increased significantly. Exosomes carry IgG bind Clq to activate the classical complement pathway to form MAC attack retinal blood vessels. This study makes up for the limitations of previous studies on exosomes in DR (106).

### Diabetic Kidney Disease (DKD)

The incidence of Diabetic kidney disease (DKD) is increasing globally, and 20-40% of diabetic patients suffer from DKD. DKD is a major cause of chronic kidney disease and end-stage kidney disease (107, 108). During the 10-20 years from hyperglycemia to renal failure, a variety of mechanisms are involved in damage to the vascular system and the glomerulus (109). Disorders of glucose and lipid metabolism, podocyte injury, basement membrane thickening, mesangial dilatation, and glomerulosclerosis are the characteristic manifestations of DKD (110, 111). The American Diabetes Association defines DKD as a progressive kidney disease related to diabetes with a glomerular filtration rate < 60 or with proteinuria (112).

Early identification and intervention can reduce and delay the progression of diabetic nephropathy into end-stage renal disease (113). There was no obvious clinical manifestation during an early-stage renal injury in diabetic patients (114). Although proteinuria is the gold standard for a renal disease diagnosis, it is affected by many factors (115). Most patients have irreversible renal damage when they have apparent clinical manifestations (116), and renal replacement therapy may be the only option once the disease progresses to end-stage renal disease (117). Accurate, early biomarkers of DKD are important.

Urine is a noninvasive body fluid that reflects kidney function (118). In previous studies, many markers (including proteins and RNA) reflecting renal function and DKD progression have been found in the urine (119). However, recent studies have found that the detection of urine exosome-related molecules is more accurately reflective of the progress of DKD (120). Exosomes are also carriers of many proteins and microRNAs in the urine (121). In the ultra-early stages of DKD, circulating exosomes cannot pass through the glomerulus to enter the urine, and the exosomes in urine are mainly derived from the kidney’s constituent cells (120). Therefore, exosome content in the urine is not easily disturbed by some peak proteins and can better mirror the progression of diabetic nephropathy.

### microRNAs

MiRNA is a small RNA with a length of about 20-24 nucleotides, which can bind to mRNA and regulate the expression of multiple target genes. It plays a vital role in immune regulation, insulin secretion, cell differentiation, and other physiological and pathological processes (122, 123). Exosomal nucleic acids exist in a remarkably stable form due to the protective effects of exosomes from RNase activity. Federica-Barutta used two-step differential centrifugation to provide the first evidence that urine exosomes contain a large amount of MiRNAs. Subsequently, many studies have confirmed that urinary microRNAs are related to the degree of lesions in DKD, which is valuable in early diagnosis and therapeutic targeting.

Researchers first examined the reported microRNAs associated with DKD in urine exosomes. MiR-215, miR-192, and miR-194 were enriched in the kidney and played a role in DKD pathogenesis (124). Yijie Jia et al. classified diabetic patients into three groups of no albuminuria, microalbuminuria, and massive albuminuria according to different albuminuria levels. They found that the levels of miR-192 in urinary exosomes was higher than the levels of miR-194 and miR-215, but miR-192 levels in the massive albuminuria group decreased. In patients with normal albuminuria and microalbuminuria, miR-192 was positively correlated with proteinuria and transforming growth factor (TGF-β). Receiver operating characteristic curve analysis shows that miR-192 is superior to miR-215 and miR-194 in distinguishing between the normal albuminuria and microalbuminuria group. MiR-192 has more predictive value in early renal function impairment in diabetic patients (125).

Yijun Xie et al. tested 5 Type 2 diabetes (T2D) patients without kidney disease and 5 T2D patients with massive proteinuria. A total of 496 types of UExo-derived miRNAs were differentially expressed in DKD patients (>2 times). After PCR verification, three up-regulated miRNAs (miR-877-3p, miR-362-3p and miR-150-5p) were found. These miRNAs are involved in the regulation of AMPK, p53 and mTOR pathways in DKD (126). Barutta F et al. evaluated miRNA expression in urine exosomes of patients with diabetes and early kidney disease. The results showed that miR-145 and miR-130a were enriched in urine exosomes of DKD patients, while miR-424 and miR-155 were reduced. In animal models of diabetic nephropathy, the levels of miR-145 in glomeruli and urine exosomes are elevated, and a high glucose environment can increase the content of miR-145 in mesangial exosomes (127).

In recent years, researchers have used bioinformatics technology to screen out further many miRNAs related to DKD. Eissa S et al. initially screened the differential expression and pathway enrichment analysis of miRNA in urine exosomes based on the syber-green PCR array. Among them, miR-15b, miR-636 and miR-34a are the three most significantly up-regulated miRNAs in DKD. Validation of qRT-PCR in larger independent subjects showed that miR-15b, miR-636 and miR-34a were all up-regulated in urine exosomes of DKD patients. It is worth noting that there is a positive correlation between these miRNAs and the ratio of serum creatinine and urine protein creatinine. The sensitivity of these urinary exosomal miRNAs detection to the diagnosis of DKD is 100% (128).

In another subsequent study, the Eissa S team used public miRNA databases to apply a combined target prediction algorithm. The PCR detection of differential expression in urine exosomes of 210 clinical participants showed that the expression levels of miR-133b, miR-30a and miR-342were higher than normal levels. A more interesting result is that there are still 39.3% miR-133b, 19.6% miR-342 and 17.9% positive rates in patients with normal albuminuria. This research indicates that these miRNAs may have changed before the patient developed albuminuria (129).

There are many other factors besides diabetes that can lead to chronic renal insufficiency. To further explore more accurate markers of DKD, Jinnan Zang et al. established a cohort study comparing urinary exosomes MiRs in patients with T2DKD and T2DM (normal renal function), as well as chronic kidney disease (CCKD). They found that let-7e-5p, miR-23b-3p and miR-21-5p were higher in T2DKD patients than CCKD patients, while the expression of miR-30b-5p and miR-125b-5p was reduced. Compared with T2DM patients in independent validation experiments, miR-21-5p was up-regulated in the T2DKD and CCKD cohorts. MiR-30b-5p has decreased expression in T2DKD and CCKD (130). Interstitial transformation of epithelial cells is a key step in renal fibrosis. MiR-21-5p in the renal cortex of diabetic mice increased inhibitory phosphatase, phosphatase and tensin homolog deleted (PTEN) and fibronectin levels (131). Overexpression of miR-21-5p promoted TGF-β-induced epithelial-mesenchymal transition (132). miR-30b-5p is abundantly expressed in prostate cancer patients, and its expression level is lower in T2DKD patients. The miR-30 family have a protective effect in kidney cells. The reduction of miR-30 family expression is also associated with renal fibrosis (133). Although the changes of miR-30b-5p and miR-21-5p are not unique to T2DKD, the degree of imbalance of miR-30b-5p and miR-21-5p in T2DKD is more obvious than that of CCKD. The definition of DKD patients is not distinguished from CCD by pathological testing methods. The different course of DKD patients is also the reason for the difficulty in finding biomarkers. How to group patients more accurately may be a breakthrough point for in-depth research in the future.

### Protein

In addition to carrying miRNAs, exosomes also contain various proteins that participate in multiple signal pathways of DKD. Irene Zubiri et al. detected 352 proteins in human urinary exosomes using proteomic techniques for the first time. Quantitative analysis revealed significant changes in 25 proteins in DKD. Further screening and analysis confirmed that there were differences in the expression of histone-lysine N-methyltransferase (MLL), α-microglobulin/bikunin precursor (AMBP), and voltage-dependent anion-selective channel protein 1 (VDAC1) (134). MLL3 is a histone methyltransferase firstly identified in exosomes (135) that is associated with proliferator-activated receptorγ (PPARγ) downstream activation. PPARγ agonists prevent a variety of kidney diseases, including DKD, through systemic and renal action (136). AMBP is a membrane glycoprotein expressed in the liver and kidneys that inhibits serine protease activity (137). Previous studies have found that compared with healthy people, patients with type 2 diabetes have lower levels of AMBP in their urine (138). The decreased synthesis of AMBP in the liver and kidneys of diabetic patients or the obstruction of exosome inclusion into AMBP may explain the decrease of urinary exosomes. VDAC 1 has been reported in exosomes secreted by both urine and B cells (139). In this study, VDAC1 levels were reduced in urine foreign bodies isolated from DKD samples (140). VDAC1 has the activity of NADH-ferricyanide reductase. In previous studies, the up-regulation of VDAC1 in the kidneys of diabetic rats was related to podocyte apoptosiss (141). The decrease in the presence of exosome VDAC1 may be related to apoptosis regulation by exosomal secretion. Due to the limitations of previous studies’ techniques and methodology, it was difficult to detect many low-abundance proteins. After removing high-abundance proteins, protein techniques can help locate potential biomarkers and therapeutic targets for DKD. This study shows us a better screening process of exosome protein markers.

Gelatinase and ceruloplasmin are two types of proteins that have been identified in urine and are closely related to the development of DKD (120, 142, 143). There is ample evidence in laboratory animals and humans that gelatinase is reduced in early and late DKD kidney tissues. The symptoms of diabetic nephropathy in mice were significantly aggravated after the deletion of gelatinase (144). Ceruloplasmin, which promotes inflammation in response to hyperglycemia and advanced glycation end products, is activated in renal tissues of patients with confirmed DKD (145). Krishnamurthy P. Gudehithlu and his team compared changes in gelatinase and ceruloplasmin levels in the urine and exosomes from DKD patients and healthy subjects. They found that the changes to gelatinase and ceruloplasmin in urinary exosomes of DKD patients were consistent with the differences in renal tissue. In contrast, the activity of these enzymes in whole urine samples from patients with DKD differed from that in renal tissues. These studies suggest that protein markers found in urinary exosomes may better reflect the kidney’s underlying changes than proteins measured in whole urine samples (120).

Aquaporins (AQPs) expressed on the plasma membrane of renal tubular epithelial cells are often dysregulated in diabetic nephropathy (146, 147). Linear regression analysis of the histological diagnosis and exosomal excretion of AQP5 and AQP2 in the urine (UAQP5 and UAQP2) of patients with non-diabetic proteinuric nephropathy (NDN) and diabetic nephropathy showed that UAQP5 and UAQP2 were positively correlated with the histological type of diabetic nephropathy (148). AQPS is a complete membrane protein. The excretion of AQP2 and other top plasma membrane proteins through exosome formation was demonstrated by immunoelectron microscopy and nano atomization liquid chromatography-tandem mass spectrometry (149). It has been suggested that exosomes can mediate the transport and secretion of cell membrane proteins in the diseased kidney.

The activation of the TGF-β/Smad is recognized as a classic signal for pathological changes of DKD (150). In recent years, various evidence has confirmed that exosomes of diabetic patients can affect many aspects of the TGF-β/Smad cascade signal. In the HG environment, the secretion of exosomes by macrophages was significantly increased. Exosomes carry TGF-β mRNA into mesangial cells, mediating the proliferation and activation of mesangial cells through the TGF-β/Smad pathway and promoting renal fibrosis (151). TGF-β superfamily receptors are classified into type I and type II receptors. TGF-β can phosphorylate Smad3 protein when it binds to type II receptor on the cell surface, regulating cell proliferation-related genes (152). The up-regulation of RII transcription is one of the mechanisms leading to the TGF-β signal activation. Akiko Sakurai et al. found that podocytes release exosomes containing the epithelial cell-specific transcription factor3 (ELF3) transcription factor in high glucose culture, promoting TGF-β/Smad signaling by up-regulating RII transcription. There was a linear correlation between the ELF3 content in exosomes and the decrease of glomerular filtration rate (153).

Podocytes are located on the surface of glomerular capillaries. They are highly differentiated epithelial cells that work with endothelium to maintain normal glomerular filtration function (154). Podocyte damage is a hallmark pathological manifestation of early DKD. Podocyte injury can lead to a variety of cellular and structural changes in the glomerulus (155). In vitro studies, exosomes released by macrophages cultured with high glucose disrupted podocyte function by inducing apoptosis and TGF-β pathway and reduced the expression of protective proteins such as nephrin, podocin and Wilms tumor protein (WT1) (156). WT1, as a podocyte-derived signal transduction factor family member, is closely related to the development and function of the urinary system (157). Clinical trials have found that the content of WT1 in urinary exosomes is related to multiple indicators that reflect the decline of kidney function (serum creatinine, albumin/creatinine ratio, urine protein/creatinine ratio and eGFR) (158). WT1 in urine exosomes is considered to be a marker of podocyte damage in DKD. These results indicate that exosomes can participate in TGF-β/Smad signal transduction in multiple pathways.

## Therapeutic Value of Exosomes

At present, the treatment of various diabetic complications aims to control the metabolic and hemodynamic changes related to this condition and slow disease progression. The treatment methods for vascular injury are very limited (159). Stem cells can self-renew and differentiate into multiple lineages to produce specific cell types (160). They are considered a potential treatment for DVC due to their ability to reconstruct damaged, lost, and aged tissues (161–163). Moreover, in recent years, research indicates that that exosomes secreted by stem cells may be an effective weapon for treating diabetic vascular diseases.

### Diabetic Retinopathy (DR) and Diabetic Kidney Disease (DKD)

A Safwat et al. injected rabbit adipose-derived mesenchymal stem cell (ADSC) exosomes into mice eyes 4, 8, and 12 weeks after the diabetic model was established by systemic, subconjunctival, and intraocular injection. They found that ADSC-derived exosomes can effectively alleviate retinal edema, the cell structure destruction in each layer in the STZ group. MiR-222 in exosomes can reduce abnormal angiogenesis by regulating STAT5a (164).

Kanna Nagaishi et al. found that after intravenous transplantation of bone marrow mesenchymal stem cells, minimal donor mesenchymal stem cells were observed in the kidneys (165). Further studies have found that bone marrow MSCs can inhibit ICAM-1 and TNF-α through paracrine exosomes and reduce the excessive infiltration of macrophages (166). At the same time, MSC-derived exosomes inhibited the mesenchymal transition and fibrosis of the renal tubular epithelial cell phenotype by reducing the expression of TGF-β (167, 168).

Autophagy has a protective effect on hyperglycemia-induced renal injury (169). Rapamycin (mTOR), the core component of cell growth signals that enhances protein translation, can inhibit autophagy when its activity is enhanced. Increased mTORC1 activity has been observed in human and animal diabetic nephropathy (170–172). Nesrine Ebrahim et al. found that bone marrow mesenchymal stem cells-derived exosomes can enhance autophagy by inhibiting the mTOR signaling pathway in the diabetic nephropathy model. Additionally, the team also found that MSC-derived exosomes significantly increased the expression of autophagy-related proteins, Beclin-1 and LC3. The histological morphology of the kidney of mice treated with MSC-derived exosomes was restored, and fibrosis markers in the kidney tissue was reduced (173).

The occurrence and development of DKD are related to podocyte injury (174). VEGF produced by podocytes is unfavorable for the treatment of DKD (175). MiR-16-5p can inhibit the expression of VEGF. Hyperglycemia reduces the production of miR-16-5p by podocytes and promotes the release of VEGF. After over-expressing MiR-16-5p in human embryonic stem cells, MiR-16-5p can be transferred to podocytes treated with high glucose through the exosomal pathway, reducing the degree of podocyte apoptosis and the expression of VEGF (176).

### Wound Blood Vessels

Approximately 15% of diabetic patients worldwide experience wound healing difficulties and diabetic foot ulcers (DFU), of which 5-24% require amputation (177). The slow reconstruction of blood vessels and inflammation make wound healing difficult in diabetic patients (178, 179). Exosomes also play an essential role in stem cell transplantation for diabetic wound angiogenesis (180). Cell-free treatment of exosomes is a highly stable, non-immune therapy that provides easy access to the lesion and has clinical value (181, 182). Evidence describes the therapeutic mechanism of MSCs-exos in diabetic wound healing. Arsalan Shabbir et al. found that MSCs-exos can be internalized by fibroblasts from normal donors and chronic wound patients and can enhance the proliferation and migration of fibroblasts in a dose-dependent manner. The uptake of MSC-exos by human umbilical vein endothelial cells also leads to increased endothelial cell lumen formation. The MSC-exos treatment activates Akt, ERK and STAT3 signaling pathways and induces the production of various growth factors (183). In another experiment, it was also confirmed that MSCs-exosomes extracted from blood accelerate wound healing in diabetic mice. MSCs-exosomes can induce macrophage polarization, enhanced angiogenesis and collagen deposition by affecting the NF-ĸB signaling pathway and up-regulation of VEGF. The expression of PTEN protein in the MSC-derived exosomes pretreated with melatonin was up-regulated, and the M2 polarization of macrophages was induced by inhibiting the phosphorylation of AKT to promote wound repair (184).

EPCs are the progenitor cells of endothelial cells, which play a crucial part in angiogenesis and diabetic wound repair (185). J Zhang et al. injected human umbilical cord blood EPC exosomes into diabetic rats and found that EPC-Exos can be integrated into endothelial cells and enhance endothelial cell proliferation, migration and blood vessel formation by activating the Erk1/2 signaling pathway (186). Chun-Yuan Chen et al. collected exosomes secreted by human urine-derived stem cells. USC-Exos protein profile screening showed that the highly expressed pro-angiogenic protein 1 (DMBT1) enhanced the angiogenic activity of endothelial cells and promoted wound healing in STZ mice (187).

Nrf2 has a protective effect on oxidative stress. Patients with Nrf2 gene mutation are more likely to have diabetic complications, including peripheral neuropathy, nephropathy, retinopathy, foot ulcer, and microvascular disease (188). Xue Li et al. used the exosomes of Nrf2 overexpressing ADSCs to treat diabetic rats and found that the foot wound ulcer area was significantly reduced. Increased levels of granulation tissue formation, angiogenesis and growth factors, as well as decreased levels of inflammatory and oxidative stress-related proteins were detected in the wound bed. This experiment shows that the target gene-modified stem cells can make their exosomes play a more effective therapeutic role (189).

As platelet-rich plasma (PRP) contains many growth factors that promote tissue regeneration and wound healing (including new blood vessel formation), PRP has been widely used to treat chronic wounds (190). Exosomes encapsulate many platelet growth factors. VEGF and bFGF carried by PRP-exos have pro-angiogenic effects in normal ECs *via* PI3K/Akt signaling (191). Wound healing begins with the proliferation of fibroblasts (192). PDGFBB promotes the proliferation of fibroblasts through the extracellular signal-regulated kinase (Erk) pathway (193). Additionally, the expression of bFGF, PDGF-BB, and TGF-β in PRP-exons was significantly enriched compared with the PRP supernatant. After PRP-exos treatment, the downstream target protein YAP of Rho GTPase (RhoA) is dephosphorylated, allowing it to transfer to the nucleus, increasing fibroblasts’ migration and promoting faster wound healing (194).

In addition to the exosomes released from stem cells, macrophages-derived exosomes also have a promising effect in mediating the pro-angiogenic effects in diabetic wounds *via* inhibiting the inflammatory response. Liu W et al. found that macrophages-derived exosomes can activate P-AKT and reduce MMP-9 levels, significantly reduce pro-inflammatory cytokines secretion, and promote the proliferation and migration of endothelial cells to improve wound healing in diabetes mellitus. Furthermore, it reduced TNF-α, IL-1β, and iNOS expression and increased the expression of IL-10 and Arg-1 (59).

After stem cell transplantation, it is unnecessary to reach the lesions for cell replacement to treat diabetic vascular damage. Exosomes may help stem cells slow down pathological changes in blood vessels throughout the body *via* blood circulation. These results have provided new suggestions for stem cell therapy.

## Conclusion and Future Perspectives

In the past diagnosis of DVC, we mainly relied on abnormal biochemical indicators produced after different organ vascular system diseases (195). However, these biochemical indicators’ values do not change significantly in the early stage of the disease and can be interfered with by many factors (196). Different types of cells can secrete exosomes. The membrane structure of exosomes prevents enzyme degradation of its contents. The outer membrane and contents of exosomes are derived from the source cell, which is a marker of pathological changes in the source cell and also a signal mediator. Many studies have confirmed that exosomes are more related to vascular complications than the signal molecules detected in the total body fluid. However, the experimenters found that the level of exosomes in the urine of patients with early diabetic nephropathy increased, the number of exosomes in the urine of end-stage diabetic nephropathy decreased (197, 198). Therefore, the influence of exosomes processing in different stages of DVC is also a challenge for converting exosomes into biomarkers.

Diabetes is a chronic disease of the whole body. Abnormal glucose metabolism and inflammation activation are the main causes of diabetic complications. Metabolic disorders in multiple organs are the main feature of diabetes. The vascular system is the most critical channel for the exchange of nutrients and signal molecules. Both paracrine and remote signals are essential ways for diabetic organs to regulate vascular disease. Exosomes connect the crosstalk between cells and blood vessels in organs and connect the regulation of blood vessels by the body’s immune system. By summarizing previous studies, we found that exosomes can activate classical pathological pathways and provide opportunities for miRNAs and microproteins to participate in diseases. It also provides new targets and perspectives for the treatment of DVC in the future.

Stem cells have always been regarded as a very promising strategy in the treatment of diabetic complications. Several studies have confirmed that stem cells can improve diabetes complications through systemic effects. In this article, we summarized the mechanism of various stem cells to treat DVC through exosomes. These results suggest that stem cells are no longer purely cell replacement therapy and are more likely to be the source of signal release for tissue repair. The treatment of stem cell exosomes will be an important direction for diabetes and vascular diseases in the future.

**Figure 2:**
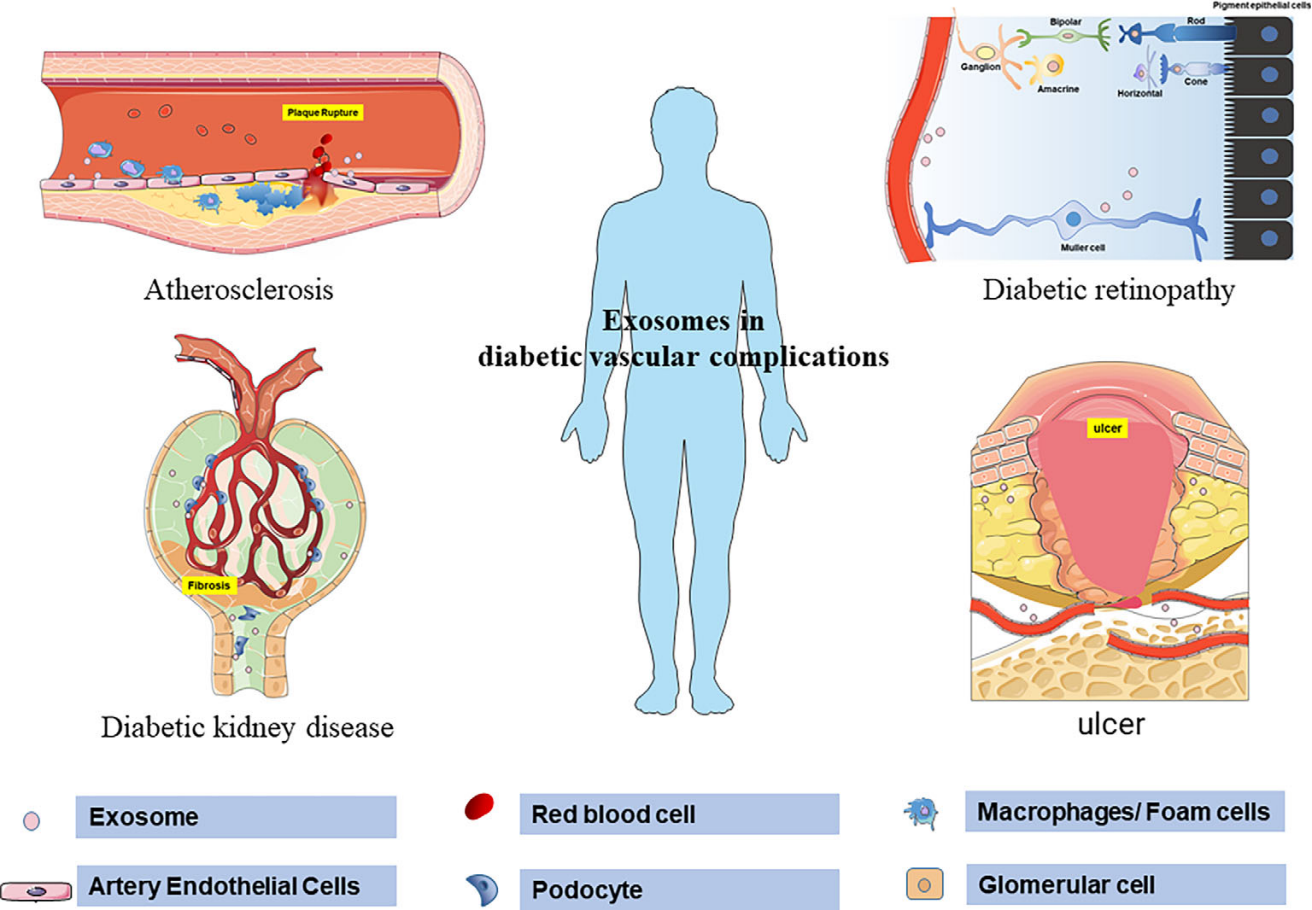
Schematic representation of exosomes regulating the pathological process of diabetic vascular complications. Exosomes mediate atherosclerosis and plaque rupture by regulating the production of no in aortic endothelium, increasing adhesion molecules, inflammatory transformation of macrophages, and endothelial proliferation in diabetes. In the retina, exosomes promote angiogenesis, destroy endothelial cells and increase leakage. At the same time, exosomes mediate the apoptosis of Müller cells in the early stage of DR and the proliferation and fibrosis of Müller cells in the late stage of DR. Exosomes carrying proteins and microRNAs mediate podocyte injury, basement membrane thickening, mesangial dilatation, and glomerulosclerosis in diabetic patients. Exosomes derived from stem cells can accelerate wound healing by regulating the proliferation and migration of fibroblasts and vascular endothelial cells.

In summary, this review emphasizes the diagnostic and therapeutic value of exosomes in DVC (**Figure 2**). However, due to the current definition of DVC, it is difficult to distinguish pathologically from similar diseases completely. The results of many exosome-related biomarker studies still need to be further confirmed. Another shortcoming in the current exosome research is the classification of the source of exosomes. Although the technology of extracting exosomes from body fluids is becoming more and more mature. However, the method of determining the source cells of exosome will be the key to pushing exosomes research to a higher level in the future. Therefore, in the study of exosomes and diabetic vascular complications, it is necessary to clarify the internal relationship between the pathological changes dominant in different stages and the changes in exosomes. In addition, exosomes from different sources represent different characteristics. By improving exosomes marking methods to clarify the distribution, uptake and half-life of exosomes, the relationship between exosomes in different cells and organs can be better clarified. It can also better avoid the non-targeted uptake and side effects of exosome therapy. In general, stem cell exosomes are a promising direction for the treatment of diabetic vascular complications, but challenges still exist in the process of clinical application in the future.

## Author Contributions

BH and YL conceived the manuscript. AC, HW, and YS wrote the manuscript. CZ and YQ draw the figure. YZ and YW wrote the table. All authors contributed to the article and approved the submitted version.

## Funding

This work was supported by National Natural Science Foundation of China (no. 82071336 to YL and no.82090044 to BH), Natural Science Foundation of Hubei Province (no. 2020CF763 to YL), National Natural Science Foundation of China (no. 81820108010 to BH, no. 81901212 to YZ, and no. 81901214 to YW), and National Key R&D Program of China (no. 2018YFC1312200).

## Conflict of Interest

The authors declare that the research was conducted in the absence of any commercial or financial relationships that could be construed as a potential conflict of interest.

## Publisher’s Note

All claims expressed in this article are solely those of the authors and do not necessarily represent those of their affiliated organizations, or those of the publisher, the editors and the reviewers. Any product that may be evaluated in this article, or claim that may be made by its manufacturer, is not guaranteed or endorsed by the publisher.
