# Supplementary material for: Exosomes: Biomarkers and Therapeutic Targets of Diabetic Vascular Complications
Source: Front Endocrinol (Lausanne). 2021 Aug 12;12:720466. doi: 10.3389/fendo.2021.720466 (PMC8387814; doi:10.3389/fendo.2021.720466)
Supplement: Supplementary file 1 [file Table_1.docx]

**Table : Contents, sources and functions of exosomes in diabetic vascular complications**

| **Types of cargo** | **RNAs/proteins Involved** | **Source** | **Disease** | **Effect** | **Ref.** |
| --- | --- | --- | --- | --- | --- |
| Protein | shh | Adipocyte | Atherosclerosis | Inducing vasa vasorum angiogenesis | [66] |
| Proteins | VCAM-1, vWF, PDGF-BB, angiopoietin-1,lysyl oxidase-2  GLUT-1, p-GP, and LAT-1 | Plasma | Atherosclerosis | Related to endothelial dysfunction | [68] |
| Protein | Arginase 1 | Blood | Atherosclerosis | Reducing NO production in endothelial cells | [74] |
| Protein | IL-1 β | coronary artery ECs | Atherosclerosis | Promoting vascular inflammation | [84] |
| MicroRNA | miR-15a | Plasma | DR | Inducing oxidative stress-mediated apoptosis of Muller cells | [97] |
| Proteins | Connective tissue growth  factor (CTGF) and fibronectin | Plasma | DR | Promoting the  proliferation and fibrosis activity of Muller cells | [98] |
| Proteins | ROS and VEGF | ARPE-19 cells | DR | Inducing abnormal blood vessel proliferation | [100] |
| MicroRNA | miR-202-5p | ARPE-19 cells | DR | Inhibiting the growth, migration and tube formation of human umbilical vein endothelial cells, and inhibiting endothelial-mesenchymal transition | [101] |
| Protein | Immunoglobulin IgG | Plasma | DR | Mediating retinal vascular injury through the classical complement pathway | [107] |
| Proteins | Gelatinase and Ceruloplasmin | Urine | DKD | Reflecting the kidney's  underlying changes | [121] |
| MicroRNA | miR-192 | Urine | DKD | Positively correlated  with albuminuria and TGF-β expression | [126] |
| MicroRNA | miR-362-3p | Urine | DKD | Mediating the  activation of the Akt/mTOR pathway in diabetic podocytes | [127] |
| MicroRNA | miR-877-3p | Urine | DKD | Leading to renal tubule cell  apoptosis | [127] |
| MicroRNA | miR-150-5p | Urine | DKD | Preventing diabetic fibrosis | [127] |
| MicroRNA | miR-145 | Urine | DKD | Involved in transforming mesangial cells | [128] |
| MicroRNAs | miR-15b, miR-34a and miR-636 | Urine | DKD | Correlated with clinical manifestations of DKD and the presence of albuminuria | [129] |
| MicroRNAs | miR-133b, miR-342, and miR-30 | Urine | DKD | Related to the negative regulation of TGFβ 1 | [130] |
| MicroRNAs | Increasing: miR-21-5p, let-7e-5p, and miR-23b-3p  Decreasing: miR-30b-5p and miR-125b-5p | Urine | DKD | Related to DKD in type 2 diabetes. | [131] |
| Proteins | AMBP,MLL3, and VDAC1 | Urine | DKD | Correlated with the kidney's  underlying changes | [135] |
| Proteins | UAQP5 and UAQP2 | Urine | DKD | Positively correlated with the histological types of DKD | [149] |
| mRNA | TGF-β1 mRNA | Macrophages | DKD | Mediating the  proliferation and activation of mesangial cells | [152] |
| Proteins | ELF3 | Podocytes | DKD | Correlated with the decrease of glomerular filtration rate | [154] |
| Protein | WT1 | Urine | DKD | Reflecting podocyte-associated glomerular dysfunction. | [159] |
| MicroRNAs | MiR-222 | ADSCs | DR | Reduce abnormal angiogenesis,edema and destroy cell structure | [165] |
| unkown | unkown | MSCs | DKD | Reduce the excessive infiltration of macrophages | [167] |
| unkown | unkown | MSCs | DKD | Increased autophagy | [174] |
| MicroRNAs | MiR-16-5p | human embryonic stem cells | DKD | Reducing podocyte apoptosis and VEGF | [177] |
| unkown | unkown | MSCs | wound | Enhance the proliferation and migration of fibroblasts | [184] |
| unkown | unkown | MSCs | wound | Induce macrophage polarization, enhanced angiogenesis and collagen deposition | [185] |
| unkown | unkown | EPCs | wound | Enhance endothelial cell proliferation, migration and blood vessel formation | [187] |
| Protein | DMBT1 | EPCs | wound | Enhanced the angiogenic activity of endothelial cells | [188] |
| Protein | Nrf2 | ADSCs | wound | Increased granulation tissue formation, angiogenesis and growth factors | [190] |
| Protein | VEGF and bFGF | Plasma | wound | Promote angiogenesis | [192] |
| Protein | bFGF, PDGF-BB and TGF-β | Plasma | wound | Proliferation of fibroblasts | [195] |
| unkown | unkown | Macrophages | wound | Reduce pro-inflammatory cytokines secretion, and promote the proliferation and migration of endothelial cells | [59] |

NO:Nitric oxide; SHH:sonic hedgehog; DR:Diabetic retinopathy; RPE:retinal pigment epithelium; HG:high-glucose; MAC: membrane attack complex; DKD:Diabetic kidney disease; MiRNAs:MicroRNAs; CTGF:connective tissue growth factor TGF-β: transforming growth factor β;T2D:Type 2 diabetes; CCKD:chronic kidney disease; PTEN:phosphatase and tensin homolog deleted; AMBP: α-microglobulin/bicunin precursor; MLL3: histone lysine N-methyltransferase; VDAC1: voltage-dependent anion-selective channel protein 1; AQPs:Aquaporins; ELF3:epithelial cell-specific transcription factor3; WT1:Wilms tumor protein; ADSCs: adipogenic mesenchymal stem cells; MSCs: mesenchymal stem cells;EPCs: progenitor cells of endothelial cells; PRP:platelet-rich plasma
